# Oral health inequalities in immigrant populations worldwide: a scoping review of dental caries and periodontal disease prevalence

**DOI:** 10.1186/s12889-024-19354-4

**Published:** 2024-07-23

**Authors:** Seyed Ahmad Banihashem Rad, Marcella Esteves-Oliveira, Anastasia Maklennan, Gail V. A. Douglas, Paolo Castiglia, Guglielmo Campus

**Affiliations:** 1 Department of Restorative, Preventive and Pediatric Dentistry, University of Bern, Freiburgstrasse 7, Bern, 3012 Switzerland; 2 Graduate School for Health Sciences, University of Bern, Bern, Switzerland; 3 Department of Restorative Dentistry and Endodontology, Justus-Liebig-University Giessen, Giessen, Germany; 4 Department of Conservative Dentistry, Periodontology and Endodontology, Oral Medicine and Maxillofacial Surgery (UZMK), University Centre of Dentistry, University of Tübingen, Tübingen, Germany; 5 Department of Dental Public Health, University of Leeds School of Dentistry, Leeds, UK; 6 Department of Medicine, Surgery and Pharmacy, University of Sassari, Sassari, Italy; 7 Department of Cariology, Saveetha Dental College and Hospitals, SIMATS, Chennai, 600077 India

**Keywords:** Global burden of oral disease, Emigrants and Immigrants, Oral health, Dental caries, Periodontal diseases, Gingivitis, DMFT, Dmft, Caries lesion

## Abstract

**Background:**

Inequalities in immigrants' oral health are often masked in population-level data. Therefore, this paper was planned to assess the prevalence data on oral health diseases, namely dental caries, and periodontitis, among immigrants worldwide.

**Methods:**

Following a systematic search in Scopus, Embase, and PubMed for studies published between 2011 and 2023, 1342 records were identified. Following title and abstract screening, 76 studies remained for full-text eligibility-screening based on predefined inclusion criteria. Thirty-two studies were included in the review.

**Results:**

Dental caries figures were higher in immigrant populations compared to the local population, regardless of host countries, age, gender, or nationality. In children, the overall mean and standard deviation (SD) for decayed, missing, and filled teeth in the primary dentition (d_3_mft) was 3.63(2.47), and for D_3_MFT (permanent dentition), it was 1.7(1.2).

Upon comparing overall mean caries counts in children and adults with their control groups in the included studies, untreated dental caries (D_3_T and d_3_t) constituted the dominant share of caries experience (D_3_MFT and d_3_mft) in immigrant children. For the local population, the highest proportion of caries experience was attributed to filled teeth (FT and ft).

Dentin caries prevalence among immigrants ranged from 22% to 88.7% in the primary dentition and 5.6% to 90.9% in the permanent dentition. Gingivitis ranged from 5.1% to 100%. Oral health varied greatly between studies. Regarding oral health accessibility, 52% to 88% of immigrant children had never been to a dentist, suggesting a very limited level of accessibility to dental health services.

**Conclusion:**

It is imperative to develop interventions and policies that have been customized to address the oral health disparities experienced by immigrant populations. Additionally, host countries should actively implement measures aimed at enhancing the accessibility of oral health care services for these individuals. The utilization of available data is crucial in establishing a hierarchy of objectives aimed at enhancing the oral health of immigrant populations.

**Trial registration:**

The Scoping review protocol was registered at OSF Registries with registration number (https://doi.org/10.17605/OSF.IO/MYXS4).

**Supplementary Information:**

The online version contains supplementary material available at 10.1186/s12889-024-19354-4.

## Introduction

In recent years, international migration has dramatically increased, becoming a significant worldwide phenomenon. According to the World Migration Report, there were 281 million international migrants in 2020 globally, an increase of 60 million from 2010. This number includes individuals of all ages who have crossed international borders to reside in countries other than their birthplace [1].

The health and oral health of immigrants may be adversely affected by a number of challenges, such as linguistic and cultural barriers, socioeconomic changes, limited access to healthcare facilities, lack of medical and dental insurance, and loss of social networks [2, 3]. These challenges can often result in poor oral health outcomes among immigrant populations. In this context, the prevalence of oral health problems is expected to be high among immigrants [4]. However, data on the extent of oral health issues and research to inform policymakers about the oral health needs of immigrants are still very limited [5]. There is an urgent need to study oral health in this population due to the growing number of immigrants.

Oral health is an important component of overall health and well-being; however, it is often overlooked in public health discussions. Oral diseases (*i.e.*, dental caries and periodontitis) contribute significantly to the global burden of chronic disease [6, 7]. These oral health conditions can cause significant pain, discomfort, tooth loss, malnutrition, and impair a person's ability to eat, communicate, and smile confidently [8, 9]. These conditions can have adverse impacts on a person's overall health and quality of life [10]. Furthermore, untreated dental caries and periodontal disease can be involved in more serious health complications, such as cardiovascular disease, respiratory infections, and even diabetes [11, 12].

With the increasing globalization and migration of people, it is important to understand the prevalence and risk factors of dental caries and periodontal problems among immigrant populations worldwide. Research has also shown that the prevalence of these oral health diseases in immigrant populations varies depending on their country of origin, level of acculturation, and length of stay in the host country [13, 14].

In summary, oral health diseases are among the most neglected aspects of health, regardless of location, culture, education, or economic standing, and particularly in low- and middle-income countries. Thus, gaining a holistic overview of the prevalence of oral health problems among immigrants might assist policymakers in defining treatment needs and treatment strategies as well as the best ways to adapt them to the health systems of the host countries. Furthermore, oral health disparities between immigrants and non-immigrants can exacerbate existing health inequities and contribute to broader health disparities.

In a previous paper, dental caries and periodontal issues in refugees were described and discussed [15]. In the present review, the focus was put on immigrant populations and compare their data with those of local population of the host country. An immigrant is someone who voluntarily relocates to a different country, whereas a refugee is an individual who is compelled to leave the country of origin.

To the best of authors’ knowledge, this is the first review that addresses dental caries and periodontal problems in the immigrant populations on a global quantitative scale. The main goals were to synthesize the evidence of the prevalence of dental caries among immigrants using the Decayed Missing and Filled index (D_3_MFT/d_3_mft) and to evaluate the prevalence of periodontal disease. Further, the dental care services provided to immigrants and their needs and deficiencies were appraised.

## Materials and methods

The Scoping review protocol was registered at OSF Registries with registration number (10.17605/OSF.IO/MYXS4). The review was completed and reported in accordance with the Preferred Reporting Items for Systematic Reviews and Meta-Analyses (PRISMA) 2020 statement [16].

### Research question and search strategy

What is the prevalence of dental caries and periodontal diseases among immigrants worldwide, and is this higher than those of the general population of the host country?

The research question for this scoping review was outlined based on sample, phenomenon of interest, design, evaluation, and research type (SPIDER) [17] tool. Three electronic databases, Scopus, Embase, and PubMed were searched using the following search strategy. Search strings were created using the keywords and synonyms in conjunction with the Boolean operators "AND" and "OR". In addition to electronic database searches, a comprehensive hand search was conducted to ensure that relevant literature was included in the review. An example of a search string used for PubMed is included here, and the others can be found in Appendix (page 2).

***S*** (Sample): (("Emigrants AND Immigrants"[MeSH Terms] OR "Undocumented Immigrants"[MeSH Terms] OR ("Refugees"[MeSH Terms] OR "Refugee Camps"[MeSH Terms]) OR "Ethnicity"[MeSH Terms] OR "Ethnic and Racial Minorities"[MeSH Terms] OR "asylum seeker*"[Title/Abstract] OR "displaced person*"[Title/Abstract] OR "refugee*"[Title/Abstract]).

***P*** (Phenomenon) of ***I*** (Interest): All the articles that related to either dental caries or periodontal problems.

***D*** (Design): not restricted.

***E*** (Evaluation): (("Dental Caries"[MeSH Terms] OR "Root Caries"[MeSH Terms] OR "Dental Caries Susceptibility"[MeSH Terms] OR "Periodontal Pocket"[MeSH Terms] OR "Periodontal Index"[MeSH Terms] OR "Gingivitis"[MeSH Terms] OR "DMF Index"[MeSH Terms] OR "dmf index*"[Title/Abstract] OR "dental decay*"[Title/Abstract] OR "carious lesion*"[Title/Abstract] OR "Carious white spot*"[Title/Abstract] OR "periodontal pocket*"[Title/Abstract] OR "dmft s*"[Title/Abstract] OR "gingival index*"[Title/Abstract] OR "dmft*"[Title/Abstract] OR "dmft index*"[Title/Abstract] OR "bleeding on probing*"[Title/Abstract] OR "probing pocket depth*"[Title/Abstract] OR "clinical attachment loss*"[Title/Abstract]).

***R*** (Research type): not restricted.

### Eligibility criteria

This scoping review included all quantitative and qualitative studies on dental caries or periodontal problems of immigrant populations of any age published from 2011 to August 2023. This timeframe was selected specifically to ensure the review is current and relevant. The review was conducted as part of a Ph.D. project addressing oral health disparities in marginalized communities. Therefore, the search population included terms like refugees and ethnic minorities, while the present review focused only on the immigrant population.

Studies with insufficient oral health data about dental caries or periodontal disease, as well as those involving refugees, asylum seekers, ethnic minorities, or indigenous populations, were excluded. Non-peer-reviewed papers and unpublished research (*e.g*., theses, abstracts, and preprints) were excluded. Only papers published in English, Italian, German, and French were considered.

### Study selection

The selection was conducted using structured procedures. After removing duplicates, the titles and abstracts of search results were examined by two independent reviewers (SABR, AM) to determine their relevance and whether they matched the planned inclusion criteria. Any uncertainties regarding the inclusion of a study were discussed with a third reviewer (GC).

### Risk of bias

After excluding ineligible papers, two independent reviewers (SABR, AM) critically rated all eligible full texts using critical appraisal instruments for prevalence studies in the Joanna Briggs Institute (JBI) System for the Unified Management of the Assessment and Review of Information (SUMARI) software (Joanna Briggs Institute, Adelaide, Australia) (appendix page 3). There were nine questions to which the answers were "yes," "no," and "unclear." Uncertainties were resolved through discussion or the assistance of a third reviewer (GC).

### Data extraction and data synthesis

One author (SABR) extracted the data using an ad hoc designed excel file for data collection, which was then checked by a second author (GC).

The following information was provided on the data extraction form:

1. Study characteristics: first author's last name, year of publication, journal, country of study, study design, sampling procedures, calculation of sample size, and methods of data collection
2. Participant characteristics and outcome measure: number of participants, sex, age, prevalence of dental caries and periodontal problems, oral health accessibility, and some other findings from the original papers.

### Parameters measured in the review

In line with the WHO methodology [18], the decayed (d_3_/D_3_), missing (m/M), and filled (f/F) teeth (d_3_mft/D_3_MFT) index score (*e.g.* DMF, DMFT, dmft, DMFS, deft, dft) was applied to evaluate oral health status [19]. Where this index is reported in this review, it refers to caries measured at the dentinal caries threshold (D_3_MF/d_3_mf) and excludes enamel caries, unless otherwise specified [20]. As we aimed to report on caries prevalence comprehensively, we included studies that utilized both WHO and ICDAS criteria. The D3/d3 level, representing caries lesions in dentine (open and closed), was chosen as a common metric. We acknowledged the differences in diagnostic thresholds between the WHO criteria, which typically focus on cavitated lesions, and the ICDAS criteria, which offer a more detailed assessment of caries progression, including non-cavitated stages. By reporting on both indices, we aimed to present a more complete picture of caries prevalence as reported in the included studies.

The mean and standard deviation (SD) of the prevalence of dental caries and range were calculated where relevant. Studies with prevalence (% d_3_mft/D_3_MFT > 0) or caries count (mean d_3_mft/D_3_MFT) data on either primary or permanent dentition or periodontal problems (*e.g.*, gingivitis, periodontitis) were taken into consideration.

Periodontal health in children and adults was evaluated using criteria such as gingivitis (Gingival Index and Community Periodontal Index), clinical attachment loss, periodontal pocket depth, bleeding on probing, and radiographic bone loss if reported by the included studies.

## Results

### Study selection

**Fig. 1:**
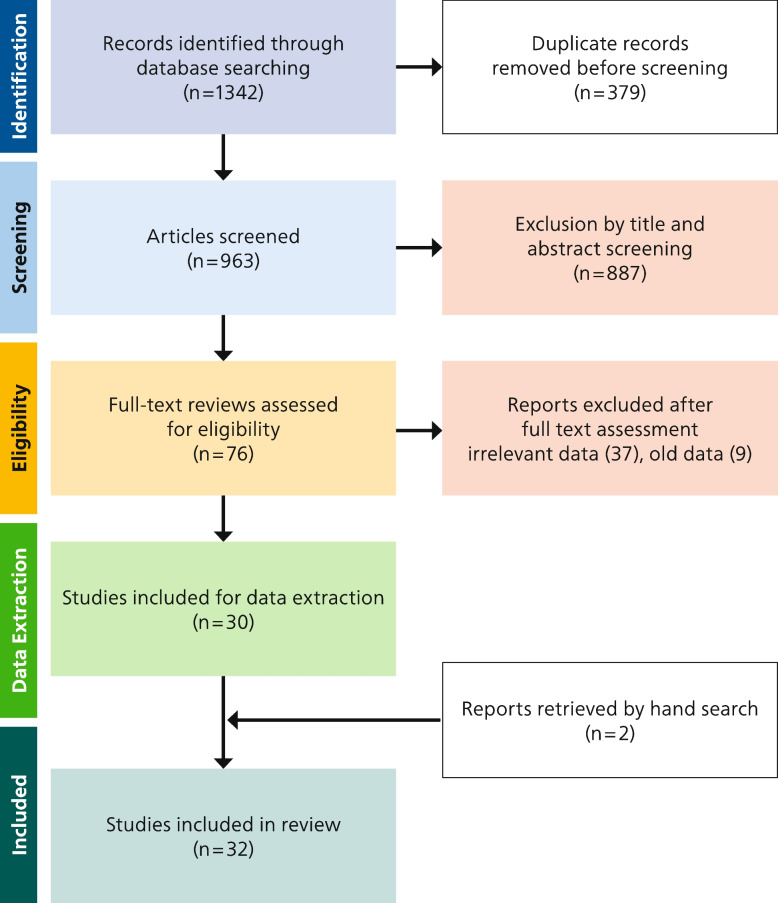
PRISMA flow diagram of study selection

The initial search with the keywords resulted in 928 papers in Scopus, 116 results in Embase, and 298 results in PubMed (Fig. 1). The authors (SABR and AM) screened the studies by title after the removal of duplicates (*n* = 379). After the title and abstract screening, 76 studies were left for full-text screening. Data extraction was then performed on 30 articles that met the inclusion criteria. In addition, two papers [21, 22] were retrieved by hand search, so overall 32 studies were included. The studies excluded after the full-text review are listed in appendix (page 4). The list of the included studies sorted by country of study is reported in Table 1.

**Table 1:** List of all included papers in the review ordered alphabetically by country where the study was conducted

	**Year of study**	**Study type**	**Country of study**	**Country of origin of study participants**	**Participants** **(n)**	**Age Range** **(years)**
Christian B et al., [23]	2012	exploratory trial	Australia	Iraq, Pakistan, and Lebanon	625	1–4
Gibbs et al., [24]	2012	Cross-sectional	Australia	Iraq, Pakistan, and Lebanon	630	1–4
Hoover et al., [25]	2012	Pilot Study	Canada	The Indian subcontinent, other parts of Asia, and the rest of the world	133	3–15
Amin et al., [26]	2013	Cross-sectional	Canada	Africa	125	1.7–6
Elyasi et al., [27]	2015	Cross-sectional	Canada	South Asia, East Asia, Africa,and East Europe	274	1–12
Dahlan et al., [28]	2017	Cross-sectional	Canada	South Asia, South East and East Asia, Arabs, Africans, East Europeans, and Hispanics	336	2–12
Azrak et al., [29]	2017	Cross-sectional	Canada	Africa, Eastern Mediterranean, and South East Asia	211	1–5.9
Liu et al., [30]	2012	Cross-sectional:	China	NR	1323	7–12
Zhang et al., [31]	2013	Cross-sectional	China	NR	10,150	5–15
Mattila et al., [32]	2012	pilot study	Finland	Iraq, Afghanistan, Iran, Russia, Thailand, Somalia, Turkey, Hungary, Slovakia, China, Vietnam, South Sudan, Syria, Sweden and Morocco	38	18–53
Aarabi et al., [33]	2012	Cross-sectional	Germany	Austria, Croatia, Italy, Turkey, Iran, Tunisia, Vietnam, Israel, Poland, Russia and Jamaica	112	60^+^
Pavlopoulou et al., [21]	2010	Cross-sectional:	Greece	Albania,Meldova,Egypt,Afghanistan, Bangladesh, India, Iran, Kenya, Lebanon, Pakistan, Ukraine,China	300	1–14
Diamanti et al., [34]	2013	Cross-sectional	Greece	Mostly Albania,Eastern European countries (such as Georgia, Romania, Bulgaria and Russia)	4409	5–15
Sivakumar et al., [35]	2016	Cross-sectional	India	Tibet	865	11–13
Ferrazzano et al., [36]	2014	Retrospective Study	Italy	NR	553	12–14
Campus et al., [37]	2017	Cross-sectional	Italy	NR	6,825	3–4
Hashizume et al., [38]	2011	Cross-sectional	Japan	Brazil	378	6–14
Lee et al., [39]	2016	Cross-sectional	South Korea	North Korea, Vietnam, China, Japan, Philippine, Thailand, Cambodia, Mongolia, and Uzbekistan	6,931	19–80
García-Pola et al., [40]	2010	prospective case–control	Spain	South America, Africa, Europe and Asia	90	6–41
Gómez-Costa et al., [41]	2011	Cross-sectional	Spain	NR	115,123	15– 64
Soria et al., [22]	2014	Cross-sectional	Spain	Morocco, Ecuador, Eastern Europe	333	6–17
Rodriguez-Alvarez et al., [42]	2016	Cross-sectional	Spain	NR	1388	4–9
Duran et al., [43]	2018	Cross-sectional	Spain	Asia, South America,Africa,Central America, North America, Euroupe	1400	3–14
Olerud et al., [44]	2014	Cross-sectional	Sweden	Iran and the Horn of Africa,Balkans,Central Asia	42	60^+^
Thorbert-Mros et al., [45]	2021	Cross-sectional	Sweden	Somalia	179	10–17
Baggio et al., [46]	2011	Cross-sectional	Switzerland	NR	856	3–6
Y.C.Lin et al., [47]	2011	Cross-sectional	Taiwan	Vietnam and Indonesia	590	4–6
Ying-Chun Lin et al., [48]	2015	Cross-sectional:	Taiwan	NR	32,611	3–5
Traisuwan et al., [49]	2016	Cross-sectional	Thailand	Myanmar,Republic of Lao,Cambodia,	418	20^+^
Meva Altas et al.¸ [50]	2022	descriptive and retrospective study	Turkey	Syria	549	6–12
Wilson et al., [51]	2013	Cross-sectional	USA	Mexico	4520	20–65^+^
Kabani et al., [52]	2011	Cross-sectional:	USA	Central and South America	9143	1–17

*NR* Not reported

### Quality assessment

No papers were excluded solely based on methodological quality assessment. Despite aiming for high methodological quality studies, we recognized that excluding moderate quality studies could potentially miss valuable insights. Studies with a quality assessment score of 5,or 6 were included, even if they weren't of the highest quality. Incorporating a broader range of evidence allowed us to gain a more comprehensive understanding of oral health disparities. Studies of moderate quality contribute valuable data and perspectives, and their inclusion helps mitigate publication bias.

Only two studies [34, 51] out of a total of 32 studies, had all the questions of the critical appraisal answered with a “yes”, gaining a score of 9 out of 9. The least favorable scores were given to questions regarding the frame and adequacy of the sample size. The lowest score was five [32, 44, 45] and four studies [25, 29, 38, 43] scored six because there was no description of the sampling frame, participant selection procedures, and sample size calculation. Only thirteen studies reported procedures for calculating sample size or if the sample size was acceptable for the target group. Nineteen studies provided a detailed description of the study's setting and participants. Four studies [35, 38, 44, 45] failed to indicate the confidence interval (CI) for the mean value. The detailed quality assessment can be found in Appendix (page 3).

### Characteristics of included studies

Seventeen studies had a control group [21, 22, 31–37, 39, 40, 42, 45, 47–50]. The control groups were the local population of the host country, except for three papers [21, 25, 32] which had a refugee population as a control group.

Among the included papers, three papers [25, 29, 47] assessed the treatment need of immigrants. Ten papers [2, 23, 26–29, 33, 38, 49, 50] reported the utilization of oral health services. Four papers [27, 30, 38, 48] investigated the dietary factors and two papers measured the household acculturation rate [28, 52]. Two papers studied the oral health status of pregnant immigrant women [40, 49] and two papers [33, 44] only included elderly population. None of the included studies had access to the oral health status of the sample group prior to their immigration.

**Fig. 2:**
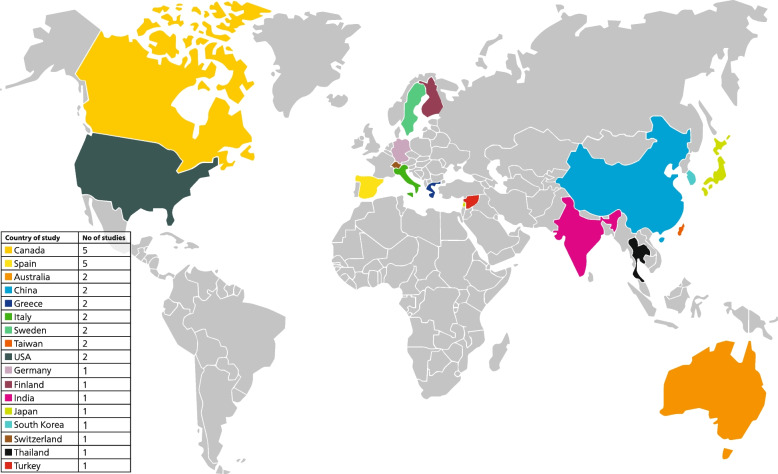
World map showing the host countries, where the studies on the oral health of immigrants have been conducted. The key on the left shows the number of studies per country, with the countries sorted by number of studies (from the highest to the lowest). Countries in which no studies could be found are marked in grey

The study participants were children in twenty-four studies, in two studies both children and adults [40, 41] and in six studies only adults [32, 33, 39, 44, 49, 51] were involved. Immigrants originated from a wide range of countries, with a majority coming from South Asia, Africa, Eastern Europe and Central and South America as listed in Table 1. The frequency and distribution of the geographical location of countries of study are shown in Fig. 2, where it is clearly observable the highest number of studies on immigrants have been conducted in Canada and Spain.

### Dental caries in immigrants

Regarding dentin caries in children, two papers [47, 48] reported higher d_3_mft counts compared to other studies included in the review (mean d_3_mft > 5), both studies were conducted in Taiwan. The overall d_3_mft count (primary dentition) of studies identified was 3.63 (2.47) and for D_3_MFT (permanent teeth), it was 1.7 (1.2). Four papers [28, 35, 36, 42] also showed an expanded version of the decayed missing filled teeth (D_3_MFT) index with individual components, as seen in Table 2.

**Table 2:** Caries distribution in immigrants and control groups in included studies^a^

	**Sample size**		**D**_**3**_**MFT**		**D**_**3**_**t**		**Mt**		**Ft**		**D**_**3**_**mft**	**d**_**3**_**t**	**mt**	**ft**	**D**_**3**_**MFT/d**_**3**_**mft**	**D**_**3**_**t/d**_**3**_**t**
**Immigrant group**																
**Adults**																
Traisuwan et al., [49]	208		5.8 (4.4)		5.5 (3.6)		1.5 (1)		3.2 (2.5)		NR	NR	NR	NR	NR	NR
García-Pola et al., [37]	45		8.33 (6.66)		NR		NR		NR		NR	NR	NR	NR	NR	NR
Aarabi et al., [33]	61		24.8 (3.9)		5.3 (4.6)		14.4 (8.8)		5 (4.6)		NR	NR	NR	NR	NR	NR
**Children**																
Hashizume et al., [38]	378		1.28 (2.22)		NR		NR		NR		NR	NR	NR	NR	NR	NR
Ferrazzano et al., [36]	183		3.92 (2.92)		2.49 (1.98)		0.88 (1.24)		0.56 (1.1)		NR	NR	NR	NR	NR	NR
Y.C. Lin et al., [47]	150		NR		NR		NR		NR		6.05	4.5	0.39	1.16	NR	NR
Liu et al., [30]	1323		2.74 (3.02)		0.01		0		NR		3.17 (3.12)	2.71	0.01	0.01	NR	NR
Sivakumar et al., [35]	431		1.14 (1.04)		1.13 (1.07)		0.04 (0.25)		0		0.18 ( 0.5)	0.12 (0.4)	0.04 (0.26)	0.02 (0.15)	NR	NR
Ying-Chun Lin et al., [48]	1046		NR		NR		NR		NR		8.47	5.38	0.3	2.79	NR	NR
Zhang et al., [31]	3412		1.05 (0.34)		NR		NR		NR		3.18 (0.57)	NR	NR	NR	NR	NR
Dahlan et al., [28]	336		NR		NR		NR		NR		NR	NR	NR	NR	3.7	NR
Diamanti et al., [34]	707		2.5 (0.14)		1.75 (0.07)		00 (0.1)		0.75 (0.21)		3 (3.8)	2.7 (3.9)	0.0 (0.4)	0.3 (1.1)	NR	NR
Rodriguez-Alvarez et al., [42]	413		0.1 (0.42)		0.1 (0.4)		NR		NR		1.7 (2.6)	1.5 (2.5)	NR	NR	NR	NR
Soria et al., [22]	177		NR		NR		NR		NR		NR	NR	NR	NR	7.8	NR
Azrak et al., [29]	211		NR		NR		NR		NR		2.2 (3.8)	1.7 (3)	0.2 (0.8)	0.3 (1.6)	NR	NR
Hoover et al., [25, 53]	44		NR		NR		0.64 (1.12)		0.48 (1.52)		NR	NR	NR	NR	3.52 (3.78)	2.41 (3.44)
Meva Altas et al.¸ [50]	549		0.94 (0.18)		NR		NR		NR		4.8 (1.6)	NR	NR	NR	NR	NR
Elyasi et al., [27]	274		NR		NR		NR		NR		NR	NR	NR	NR	3.28 (3.76)	NR
García-Pola et al., [37]	45		NR		NR		NR		NR		NR	3.5 (3.4)	NR	NR	NR	NR
Overall	9679		1.7 (1·2)		1.36 (1.01)		0.52 (0.43)		0.59 (0.13)		3.63 (2.47)	2.76 (1.69)	0.18 (0.16)	0.76 (1.07)	4.57 (2.15)	2.41
**Control group**																
**Adults**																
Traisuwan et al., [49]	210	4.8 (4)		3.8 (2.9)		2 (1.5)		3.1 (2.5)		NR		NR	NR	NR	NR	NR
García-Pola et al., [37]	45	8.07 (6.05)		NR		NR		NR		NR		NR	NR	NR	NR	NR
Aarabi et al., [33]	51	23.4 (4.6)		2.1 (2.8)		12.6 (9.5)		8.6 (6.2)		NR		NR	NR	NR	NR	NR
**Children**																
Y.C.Lin et al., [47]	440	NR		NR		NR		NR		3.88		1.57	0.17	2.13	NR	NR
Ferrazzano et al., [36]	370	3.29 (3.21)		1.16 (1.35)		0.71 (1.43)		1.38 (1.98)		NR		NR	NR	NR	NR	NR
Sivakumar et al., [35]	434	0.45 (0.8)		0.32 (0.69)		0.02 (0.16)		0.1 (0.36)		0.58 (0.98)		0.3 (0.72)	0.23 (0.65)	0.04 (0.21)	NR	NR
Ying-Chun Lin et al., [48]	31,565	NR		NR		NR		NR		8.10		4.37	0.23	3.5	NR	NR
Zhang et al., [31]	6738	1 (0.31)		NR		NR		NR		2.61 (0.66)		NR	NR	NR	NR	NR
Rodriguez-Alvarez et al., [42]	839	0.0 (0.28)		0.0 (0.2)		NR		NR		0.7 (1.5)		0.6 (1.4)	NR	NR	NR	NR
Soria et al., [22]	136	NR		NR		NR		NR		NR		NR	NR	NR	6.6	7.3 (4.4)
Hoover et al., [25]	89	NR		NR		1.25 (2.2)		1.55 (2.36)		NR		NR	NR	NR	5.8 (4.2)	3 (3.4)
García-Pola et al., [37]	45	NR		NR		NR		NR		NR		0.24 (0.6)	NR	NR	NR	NR
Overall	40,656	1.58 (1.5)		0.74 (0.59)		0.66 (0.61)		1.01 (0.79)		2.68 (3)		1.71 (1.85)	0.21 (0.03)	1.89 (1.74)	6.2 (0.56)	5.15 (3.04)

D_3_MFT – caries experience in the permanent dentition, d_3_mft – caries experience in the primary dentition, D_3_T – decayed teeth in the permanent dentition, MT – missing teeth in the permanent dentition, FT – filled teeth in the permanent dentition, d_3_t – decayed teeth in the primary dentition, mt – missing teeth in the primary dentition, ft – filled teeth in the primary dentition, *NR* Not reported, *SD* Standard deviation

^a^Data presented as mean (SD) unless otherwise specified

Upon comparing the overall caries means of the included studies, untreated dental Caries (D_3_T and d_3_t) constituted the dominant share of the caries experience (D_3_MFT and d_3_mft) in immigrant children. While, within their respective control groups, the highest proportion of caries experience was attributed to Filled Teeth (FT and ft).

Among the papers that had the local population as control group, the immigrant children had a higher mean D_3_MFT/d_3_mft (SD) compared to local children. This difference was significant except for two papers [31, 42], which only showed a significant difference for primary dentition and not the permanent dentition.

There were only three studies [33, 40, 49] reported caries using D_3_MFT in adults, suggesting that there is a lack of caries data in immigrant adults. The mean D_3_MFT count among immigrant adults was higher than that of the local population. This difference was significant except for two studies [33, 40]. It is important to emphasize that we only reported the statistics generated by the included studies. As regards caries experience, due to the limited number of studies and heterogeneity of study participants in the adult population, the overall mean for caries experience was not calculated.

### Caries prevalence and further detail of included papers

The main focus of all included studies was oral health (OH) except for two [21, 25], which also involved general health (GH). Only three studies [21, 42] reported a caries prevalence of below 20% for immigrant children. Caries prevalence in the primary dentition ranged from 22% to 88.7%, and in the permanent dentition from 5.6% to 90.9%. Overall, the caries prevalence, regardless of dentition stage, ranged from 17% to 97.3% among the immigrant population (Table 3).

**Table 3:** Further detail of included papers and caries prevalence

	**Focus GH or OH**	**Dentist involved**	**Instruments mentioned**	**Reliability tested†**	**Caries detection method**	**Caries prevalence** **(%)**	**Caries prevalence in control group** **(%)**
**Primary Dentition**							
Christian B et al., [23]	OH	NR	NR	NR	ICDAS II	22	NR
Gibbs et al., [24]	OH	Yes	Yes	Yes	ICDAS/ WHO	34	NR
Baggio et al., [46]	OH	Yes	Yes	Yes	WHO	38.6	12.1
Rodriguez-Alvarez et al., [42]	OH	One dentist	Yes	No	WHO	42.6	24.1
Azrak et al., [29]	OH	Yes	Yes	NR	WHO	45.5	NR
Amin et al., [26]	OH	Yes	Yes	Yes	WHO	56	NR
Duran et al., [43]	OH	Yes	Yes	Yes	NR	62.3	42.6
Diamanti et al., [34]	OH	Yes	Yes	Yes	ICDAS II	64.2	NR
Liu et al., [30]	OH	Yes	Yes	Yes	WHO	65.7	NR
García-Pola et al., [37]	OH	Yes	Yes	NR	WHO	66.6	15.5
Ying-Chun Lin et al., [48]	OH	Yes	Yes	NR	WHO	68.1	56.7
Zhang et al., [31]	OH	Yes	Yes	Yes	WHO	71.4	64.5
Campus et al., [37]	OH	Yes	Yes	Yes	ICDAS	72.6	41.6
Y.C. Lin et al., [47]	OH	NR	Yes	Yes	WHO	88.7	NR
**Permanent Dentition**							
Rodriguez-Alvarez et al., [42]	OH	One dentist	Yes	No	WHO	5.6	2.4
Duran et al., [43]	OH	Yes	Yes	Yes	NR	16.4	12.2
Liu et al., [30]	OH	Yes	Yes	Yes	WHO	28.1	NR
Wilson et al., [51]	OH	Yes	NR	NR	NS	38	34.4
Hashizume et al., [38]	OH	Yes	Yes	NR	WHO	38.1	NR
Zhang et al., [31]	OH	Yes	Yes	Yes	WHO	42.5	39.6
Lee et al., [39]	OH	Yes	Yes	NR	WHO	54.8	24.9
Mattila et al., [32]	OH	Yes	NR	NR	NR	65	57
Diamanti et al., [34]	OH	Yes	Yes	Yes	WHO	67.1,	NR
Sivakumar et al., [35]	OH	NR	Yes	Yes	WHO	71	53.9
Olerud et al., [44]	OH	One dentist	Yes	NR	NR	75	NR
Ferrazzano et al., [36]	OH	Yes	Yes	Yes	WHO	77.5	55.9
García-Pola et al., [37]	OH	Yes	Yes	NR	WHO	88.9	80
Traisuwan et al., [49]	OH	Yes	NR	Yes	WHO	90.9	85.2
Aarabi et al., [33]	OH	Yes	Yes	Yes	WHO	NR	NR
**Unspecified dentition**							
Pavlopoulou et al., [21]	GH	NR	NR	NR	NR	17.4	24.7
Kabani et al., [52]	OH	NR	NR	NR	WHO	24.9	NR
Elyasi et al., [27]	OH	Yes	Yes	NR	WHO	52	NR
Soria et al., [22]	OH	Yes	NR	NR	NR	92.3	NR
Meva Altas et al.¸ [50]	OH	One dentist	Yes	NR	NR	97.3	NR
Dahlan et al., [28]	OH	Yes	Yes	NR	WHO	NR	NR
Gómez-Costa et al., [41]	OH	Yes	NR	NR	WHO	NR	NR
Thorbert-Mros et al., [45]	OH	Yes	Yes	NR	WHO	NR	NR
Hoover et al., [25]	GH	Yes	Yes	NR	NR	NR	NR

*GH* General health, *OH* Oral health, *NR* Not reported, *WHO* World health organization, *ICDAS* International Caries Detection and Assessment System

†Reliability tested: If the studies gave information about inter or intra reliability of dental examination, it is showed as Yes or NR. The studies that did not report the caries prevalence, reported caries in other forms DMFT/S

When comparing the caries prevalence to the local population, the immigrants always had a higher prevalence. Only one study [21] reported a lower caries prevalence than in the control group however, in this instance the control group was a refugee population. Visual comparison of caries prevalence between immigrant groups and their corresponding control groups via bar charts can be found in the appendix (page 5).

### Other indices to report caries: DMFS and ICDAS

Five papers [24, 26, 29, 34, 37] reported caries prevalence in other forms using D_3_MF at the surface level (D_3_MFS) or International Caries Detection and Assessment System* (*ICDAS). Two papers [26, 29] reported caries using D_3_MFS (Table 4). Two papers [24, 34] used the dmfs index derived from the full range of ICDAS scores [53], as a result, their count of caries experience included both enamel and dentine caries since both are recorded by the ICDAS index [54]. Analysis of tooth surfaces found that early caries lesions were especially frequent in age groups 12 and 15, with respective mean values of 1.9(2.1) and 2.4(3.0) [34].

**Table 4:** Caries distribution in immigrants in studies using DMFS and ICDAS as caries indices^a^

	**Sample size**	**Age in years**	**dmfs**	**Decayed surfaces**	**Missing surfaces**	**Filled surfaces**
Amin et al., [26]	125	1.7–6	7.2 (11.6)	4.2 (7.4)	NR	NR
Azrak et al., [29]	211	1–5.9	4.8 (11)	3 (6.7)	0.7 (3.5)	1.1 (6.2)
***ICDAS Study***	**Sample size**	**Age in years**	**DMFS/dmfs **_**ICDAS 1–6**_	**DFMS/dmfs **_**ICDAS1-3**_		**DMFS/dmfs **_**ICDAS 4–6**_
Diamanti et al., [34]	707	5	4.1 (9.1)	1.1 (1.6)		5 (7.9)
		12	3.6 (4.4)	1.9 (2.1)		3.6 (4.4)
		15	3.7 (4.8)	2.4 (3)		3.7 (4.8)
Gibbs et al., [24]	630	1–4	1.9 (4.62)	NA		0.91 (3.47)

*NR* Not reported, *DMFS* Decayed, missing, and filled surfaces, *ICDAS* International Caries Detection and Assessment System

^a^Caries value is reported as mean (SD) unless otherwise specified

### Periodontal Health in immigrants

Nine papers [25, 32, 33, 41, 44, 45, 49–51] examined the periodontal health. Four of them [25, 32, 45, 50] focused on children and five [33, 41, 44, 49, 51] on adults. Two paper [33, 44] only included an elderly population and one paper included only pregnant migrant women [49].

Regarding periodontal health in children, the prevalence of gingivitis ranged from 5.1% to 100%, indicating a high variation. In particular, the prevalence of gingivitis was reported as very high in three studies [25, 32, 45], with one paper reporting that almost all children had chronic gingivitis [45] and two papers reporting a prevalence of two thirds [25, 32]. Although gingival inflammation was apparently high from the aforementioned studies, one paper [50] reported a prevalence of gingivitis of 5.1%. Another paper showed a higher prevalence of gingivitis in immigrant children compared to the local population with a margin of 25% [45].

Regarding periodontal health in adults, the prevalence of periodontitis was present in half of the population observed [51], similar was observed in another study [44] which reported two-thirds of participants had periodontitis and a quarter of them were diagnosed with severe periodontitis (gingival pockets of 6 mm or deeper). Based on the Papillary Bleeding Index, a study [33] conducted on elderly immigrants showed a greater prevalence of papillary bleeding compared to their peers (46.3% *vs* 30.5%).

The one paper that included only pregnant Immigrant women, reported almost all participants had gingivitis, the periodontitis was three times more prevalent in immigrant pregnant women compared to local pregnant women (74.5% vs 22.4%). Moreover, 11% were diagnosed with severe periodontitis compared to only 0.5% in the host population, which showed a huge difference in periodontal health between pregnant migrant women and local pregnant ones [49].

### Oral health accessibility

Access to oral health care is an important determinant of oral health status [55]. Unfamiliarity with the dental care delivery system, lack of proper insurance (where relevant) and high costs of dental treatment might make obtaining proper oral care difficult [26].

Eight papers [23, 26–29, 33, 49, 50] explored the history of dental visits in immigrants, all papers addressed children except for two [33, 49]. Four papers [27, 28, 33, 49] reported, whether the participants have had a dental visit in the last year while others asked about history of dental visit in their lifetime.

When asking immigrants’ children about the history of their last dentist visit, the percentage of children who never visited a dentist in their life, ranged from 52 to 88% (appendix page 6). For adults, there was a significant difference in dental visits between migrants and local women, with 61.1% of migrants never having visited the dentist or visiting less frequently than once a year [49]. Regarding last year dental visit, 88.2% of non-migrant Germans had at least one dental examination, compared to 68.9% of immigrants.

## Discussion

Based on the included studies, it was evident that immigrants were more likely to suffer from oral health problems than the local population in their host country. The perceived treatment needs varied between studies, still dental caries and periodontal disease were most commonly regarded as urgent problems among immigrants.

A variety of factors have been identified as influencing dental caries prevalence among immigrant children, including family socioeconomic status, household acculturation, oral health accessibility, child's age, gingival inflammation, fluoride exposure,country of origin, and generational status [22, 25, 46, 52]. These factors collectively contribute to caries development, highlighting the complex interaction between diverse influences on dental health outcomes within different demographic contexts.

Acculturation and oral health have a dynamic relationship [3]. Oral health might be affected by acculturation, which has been defined as "lifestyle and behavioral changes as a result of moving from one culture to another, usually as a result of immigration” [56]. According to one study [52], household acculturation was a significant predictor of dental caries in children, whereas another study [28] found no association between parental acculturation and children’s dmft/DMFT level.

Lower age was directly correlated to higher caries prevalence [34, 50]. In another study, the same was observed but just for the primary dentition [31]. The disparity in caries between immigrant children and their peers in older age groups was less, which it has been suggested indicates that the dental health of migrants children was better in older children [31, 34, 50]. The decrease in caries disparity among older immigrant children might be due to improved socioeconomic status of parents [34], increased access to oral health services, local peers’ influences at schools [31], and ultimately development of better oral health habits, such as proper oral hygiene practices(frequent and adequate brushing and flossing) and healthier nutritional choices. It might be hypothesized that the older children are more mature and generally more familiarized with the new language and therefore adopt easier to dental health habits of their host country, while younger children usually continue to follow their parents' traditional practices. According to a study conducted in Spain, the second generation of immigrant children had lower caries prevalence than first-generation and they were almost similar to Spanish-born children after adjustment for confounders (social class, marital status, and maternal education) [22]. However, there are many confounding variables at play, as well as methodological limitations, which limits confidence in any conclusions about age-related disparities drawn from cross-sectional studies.

All studies, except one [41], reported that the prevalence of periodontitis in immigrants was higher compared to the local population. According to one study [41], there was a similar proportion of gum bleeding among immigrants (16 to 23%) and Spanish nationals (17 to 21%). immigrant women, as well as immigrants between the ages of 25–64, were less likely to experience gum bleeding than their local peers.

The socio-demographic characteristics of immigrant children significantly impacted their use of dental care. These factors included parental education [28], income level [28], dental coverage [23, 26–28], child's age [26], mother's age [26, 28], the duration of parental residence in the host country [26], household structure [28] (whether living with both parents or with a single parent), frequency of parent's dental visits [23] (characterized by infrequent attendance), primary reasons for dentist visits (primarily for treatment rather than preventive care) [23], parental perception of the child's dental care needs [23], and parental assimilation scores [28]. Among the various factors considered by the studies, requiring insurance coverage was identified as the most common and significant factor affecting children's dental visits [23, 26–28]. One paper [23] specifically explores reasons related to the immigrant child’s non-utilization of dental services and their parents/guardians reported cost, long waiting periods for treatment, language barriers and “no need for child to visit” were the main barriers for accessing to oral health services for their children.

Oral health disparities are not limited to immigrant groups and are widespread in numerous nations, reflecting the present global tendency to emphasize specialized treatments rather than ensuring equal access to care [57, 58]. It is evident that a number of global factors might be contributing to the weaker oral health of immigrants compared to native people in host countries. Firstly, there are disparities in oral health across the world that are impacted by socioeconomic, cultural, and environmental variables. Second, these difficulties could also be exacerbated by obstacles such as language barriers, inadequate insurance, and unfamiliarity with the healthcare systems in the host countries. Oral health disparities across immigrant populations can be made exacerbated by differences in income, education, and healthcare facilities between the countries of origin and the host countries. To address these global factors contributing to oral health inequalities, multi-level interventions aimed at providing equitable access to dental healthcare services are needed [57, 59].

Our search strategy was unable to find any studies conducted in South America or Africa. This might be attributed to a lack of scientific research on immigrant dental caries or periodontal problems after 2011 in these regions or to the fact that these studies have not yet been published in indexed journals. In our review, the majority of studies employed cross-sectional designs and had a pure descriptive scope, indicating that this issue is still in its exploratory phase.

This scoping review has some limitations, including the possibility that some information could have been overlooked, as the studies retrieved in the systematic database search showed considerable differences in the characterization and reported data of the immigrant population. Additionally, we observed significant differences in sampling procedures, power calculations, and geographic location among the included studies; some studies [25, 29, 38, 43] did not specify sample size calculation, and immigrant populations were generally smaller than the control groups. There are also existing intra-immigrant disparities, which might be due to variations in socioeconomic status, healthcare access, cultural practices, and health literacy which was not discussed in detail in our review.

Due to a lack of comparability and high heterogeneity among the studies, we did not conduct a meta-analysis. Since we included studies published exclusively after 2011, our findings are less generalizable due to the limited number of publications on this topic, especially from developing and underdeveloped countries. Moreover, including only articles published in English, Italian, German, and French might have introduced a language bias, excluding studies published in other languages. In addition, human errors and bias may have contributed to the loss of information or bias of the results.

In spite of these limitations, to our knowledge, the present review was the first to summarize oral health diseases of immigrants in a quantitative manner on a global scale. The study provides additional information on special needs and associations that can be used to improve oral health in immigrants.

The findings of our study have significant implications for professionals in oral health as well as public health efforts. Inequalities in immigrants' oral health care are often masked by population-level data since immigrants constitute a small proportion of populations in host countries. Our findings successfully addressed the reality of immigrant oral health in their respective countries.

## Conclusion

There is a higher prevalence of dental caries among immigrants than among the local population in each host country, regardless of age, gender, or country. Untreated dental caries (D_3_T, d3t) were more prevalent in this population. The existing data can be used to set priorities for improving immigrants' oral health worldwide. Immigrants worldwide face major oral health challenges, including dental caries, periodontal diseases and limited access to oral health services.

Efforts must be made to reduce oral health disparities among immigrants. Host countries must implement strategies to significantly increase access to dental care for immigrants such as Providing oral health insurance to immigrant children, developing community healthcare centers, expanding financial assistance, and integrating dental services into primary healthcare.. Further studies are needed to contribute to real-world knowledge about immigrants' oral health, as they can assist host-country policymakers in improving immigrants' oral health and developing more cost-effective preventative measures.

### Supplementary Information

Supplementary Material 1.

## Data Availability

Any data that support the findings of this study are available from the corresponding author, upon reasonable request.

## Abbreviations

OSF: Open science framework
PRISMA: Preferred Reporting Items for Systematic Reviews and Meta-Analyses
SPIDER: Sample phenomenon of interest, design, evaluation, and research type
JBI SUMARI: Joanna Briggs Institute System for the Unified Management of the Assessment and Review of Information
WHO: World health organization
D_3_MFT: Caries experience in the permanent dentition
d_3_mft: Caries experience in the primary dentition
D_3_T: Decayed teeth in the permanent dentition
MT: Missing teeth in the permanent dentition
FT: Filled teeth in the permanent dentition
d_3_t: Decayed teeth in the primary dentition
mt: Missing teeth in the primary dentition
ft: Filled teeth in the primary dentition
NR: Not reported
SD: Standard deviation
GH: General health
OH: Oral health
ICDAS: International Caries Detection and Assessment System
DMFS: Decayed, missing, and filled surfaces
