# Supplementary material for: Oral health inequalities in immigrant populations worldwide: a scoping review of dental caries and periodontal disease prevalence
Source: BMC Public Health. 2024 Jul 23;24:1968. doi: 10.1186/s12889-024-19354-4 (PMC11267954; doi:10.1186/s12889-024-19354-4)
Supplement: Supplementary file 1 — Supplementary Material 1. [file 12889_2024_19354_MOESM1_ESM.docx]

**Supplementary files for “Oral Health Inequalities in Immigrant Populations Worldwide: A Scoping Review of Dental Caries and Periodontal Disease Prevalence”**

**CONTENTS**

[Search strategy for each electronic database 2](#_Toc99034649)

Risk of bias assessment [3](#_Toc99034650)

[Excluded studies after the assessment of full texts and reasons for exclusion (N=46). 4](#_Toc99034651)

Comparison of caries prevalence between immigrant and control groups divided by dentition stage 5

[History of dental visits in immigrants 6](#_Toc99034655)

[References of excluded studies 6](#_Toc99034655)

**Appendix 1. Detailed search procedure in databases**

| **Pubmed** (n=298) | (("Emigrants AND Immigrants"[MeSH Terms] OR "Undocumented Immigrants"[MeSH Terms] OR ("Refugees"[MeSH Terms] OR "Refugee Camps"[MeSH Terms]) OR "Ethnicity"[MeSH Terms] OR "Ethnic and Racial Minorities"[MeSH Terms] OR "asylum seeker*"[Title/Abstract] OR "displaced person*"[Title/Abstract] OR "refugee*"[Title/Abstract]) AND ("Dental Caries"[MeSH Terms] OR "Root Caries"[MeSH Terms] OR "Dental Caries Susceptibility"[MeSH Terms] OR "Periodontal Pocket"[MeSH Terms] OR "Periodontal Index"[MeSH Terms] OR "Gingivitis"[MeSH Terms] OR "DMF Index"[MeSH Terms] OR "dmf index*"[Title/Abstract] OR "dental decay*"[Title/Abstract] OR "carious lesion*"[Title/Abstract] OR "carious white spot*"[Title/Abstract] OR "periodontal pocket*"[Title/Abstract] OR "dmft s*"[Title/Abstract] OR "gingival index*"[Title/Abstract] OR "dmft*"[Title/Abstract] OR "dmft index*"[Title/Abstract] OR "bleeding on probing*"[Title/Abstract] OR "probing pocket depth*"[Title/Abstract] OR "clinical attachment loss*"[Title/Abstract]) AND (("all"[Filter] NOT "preprint"[Publication Type]) AND ("english"[Language] OR "french"[Language] OR "italian"[Language]) AND 2011/01/01:2023/12/31[Date - Publication])) AND ((excludepreprints[Filter]) AND (english[Filter] OR french[Filter] OR german[Filter] OR italian[Filter]) AND (2011:2023[pdat])) |
| --- | --- |
| **EMBASE** (n=116) | Query('immigrant':ti,ab OR 'immigrants':ti,ab OR 'refugee':ti,ab OR 'refugee camp':ab OR 'asylum seeker':ti,ab OR 'ethnic group':ti,ab) AND ('dental caries':ti,ab OR 'dmf index':ti,ab OR 'dmft index':ti,ab OR 'dmfs index':ti,ab OR 'caries assessment':ti,ab OR 'periodontal disease':ti,ab OR 'gingiva disease':ti,ab OR 'gingivitis':ti,ab OR 'periodontal pocket depth':ti,ab OR 'pocket depth':ti,ab OR 'gingival index':ti,ab OR 'bleeding on probing':ti,ab OR 'clinical attachment level':ti,ab OR 'decay rate':ti,ab) AND (2011:py OR 2012:py OR 2013:py OR 2014:py OR 2015:py OR 2016:py OR 2017:py OR 2018:py OR 2019:py OR 2020:py OR 2021:py OR 2022:py OR 2023:py) AND ([english]/lim OR [french]/lim OR [german]/lim OR [italian]/lim) AND ([embase]/lim OR [medline]/lim OR [pubmed-not-medline]/lim) |
| **Scopus** (n=928) | ( TITLE-ABS-KEY ( immigrant* OR refugee* OR "refugee camp" OR "asylum seeker" OR "ethnic group" ) AND ALL ( ( "dental caries" OR "DMF index" OR "DMFT index" OR "DMFS index" OR "caries" OR "periodontal disease" OR "gingiva disease" OR "gingivitis" OR "periodontal pocket depth" OR "pocket depth" OR "gingival index" OR "bleeding on probing" OR "clinical attachment level" OR "decay rate" ) ) ) AND PUBYEAR > 2010 AND PUBYEAR < 2024 AND ( LIMIT-TO ( EXACTKEYWORD , "Human" ) ) AND ( LIMIT-TO ( LANGUAGE , "English" ) OR LIMIT-TO ( LANGUAGE , "German" ) OR LIMIT-TO ( LANGUAGE , "French" ) ) OR LIMIT-TO ( LANGUAGE , "Italian" ) ) |

**Appendix 2: Quality assessment (JBI SUMARI)**

| Citation | Q1 | Q2 | Q3 | Q4 | Q5 | Q6 | Q7 | Q8 | Q9 | Total score |
| --- | --- | --- | --- | --- | --- | --- | --- | --- | --- | --- |
| Christian B et al,(23) | Y | U | Y | Y | Y | Y | Y | N | Y | 7 |
| Gibbs et al,(24) | Y | U | Y | Y | Y | Y | Y | Y | Y | 7 |
| Hoover et al,(25) | N | Y | N | Y | U | Y | Y | Y | Y | 6 |
| Amin et al,(26) | Y | Y | N | Y | Y | Y | Y | Y | U | 7 |
| Elyasi et al,(27) | U | Y | U | Y | Y | Y | Y | Y | Y | 7 |
| Dahlan et al,(28) | N | Y | N | Y | Y | Y | Y | Y | Y | 7 |
| Azrak et al,(29) | N | U | N | Y | Y | Y | U | Y | Y | 6 |
| Liu et al,(30) | Y | Y | Y | U | Y | Y | Y | Y | Y | 8 |
| Zhang et al,(31) | Y | Y | Y | U | Y | Y | Y | Y | Y | 8 |
| Mattila et al,(32) | N | U | N | Y | Y | Y | U | Y | Y | 5 |
| Aarabi et al,(33) | U | Y | U | Y | Y | Y | Y | Y | Y | 7 |
| Pavlopoulou et al,(21) | U | Y | U | Y | Y | Y | Y | Y | Y | 8 |
| Diamanti et al, (34) | Y | Y | Y | Y | Y | Y | Y | Y | Y | 9 |
| Sivakumar et al,(35) | U | Y | Y | Y | Y | Y | Y | U | Y | 7 |
| Ferrazzano et al,(36) | Y | Y | Y | U | Y | Y | Y | Y | Y | 7 |
| Campus et al,(37) | Y | Y | Y | U | Y | Y | Y | Y | Y | 8 |
| Hashizume et al,(38) | U | Y | N | Y | N | Y | Y | Y | Y | 6 |
| Lee et al,(39) | Y | Y | Y | U | Y | Y | Y | Y | Y | 8 |
| García-Pola et al,(40) | Y | Y | U | Y | U | Y | Y | Y | Y | 7 |
| Gómez‑Costa et al,(41) | Y | Y | Y | U | Y | Y | Y | Y | Y | 8 |
| Soria et al,(22) | N | Y | N | Y | Y | Y | Y | Y | Y | 7 |
| Rodriguez-Alvarez et al,(42) | Y | Y | Y | U | Y | Y | Y | Y | Y | 8 |
| Duran et al,(43) | Y | Y | Y | Y | U | U | U | Y | Y | 6 |
| Olerud et al,(44) | N | Y | N | Y | Y | Y | Y | U | U | 5 |
| Thorbert-Mros et al,(45) | N | Y | N | Y | Y | Y | Y | U | U | 5 |
| Baggio et al,(46) | Y | Y | Y | U | Y | Y | Y | Y | Y | 8 |
| Y.C. Lin et al,(47) | Y | Y | U | Y | Y | Y | Y | Y | Y | 8 |
| Ying-Chun Lin et al,(48) | Y | Y | Y | U | Y | Y | Y | Y | Y | 8 |
| Traisuwan et al,(49) | Y | Y | U | Y | Y | Y | y | Y | Y | 8 |
| Meva Altas et al¸(50) | Y | U | Y | Y | Y | Y | Y | Y | Y | 8 |
| Wilson et al,(51) | Y | Y | Y | Y | Y | Y | Y | Y | Y | 9 |
| Kabani et al,(52) | Y | Y | Y | Y | Y | U | Y | Y | Y | 8 |
| Total (%) yes | 64.5 | 87 | 54.9 | 74.2 | 90.3 | 96.7 | 93.5 | 89 | 93.3 |  |
| Critical appraisal questions: Q1. Was the frame appropriate to address the target population? Q2. Were study participants sampled in an appropriate way? Q3. Was the sample size adequate? Q4. Were the study subjects and the setting described in detail? Q5. Was data analysis conducted with sufficient coverage of the identified sample? Q6. Were valid methods used for identification of the condition? Q7. Was the condition measured in a standard, reliable way for all participants? Q8. Was there appropriate statistical analysis? Q9. Was the response rate adequate and, if not, was the low response rate managed appropriately? Y, yes; U, unclear; N, no | | | | | | | | | | |

**Appendix 3: Excluded studies (n=46)**

| **Excluded Studies** | **REASON FOR EXCLUSION** |
| --- | --- |
| 1. Nicol et al,(2015) | The study participants were refugees. |
| 1. Marwaha et al(2022), | The study participants were refugees. |
| 1. Moreau et al, (2019) | The study participants were refugees. |
| 1. van Berlaer G, (2016) | The study participants were refugees. |
| 1. Goetz et al,(2018) | The study participants were refugees. |
| 1. Solyman et al,(2018) | The study participants were refugees. |
| 1. Takriti et al, (2021) | The study participants were refugees. |
| 1. Al-Ani et al, (2016) | The study participants were refugees. |
| 1. Kakalou et al, (2018) | The study participants were refugees. |
| 1. Bhatt et al, (2019) | The study participants were refugees. |
| 1. Noaman et al,(2019) | The study participants were refugees. |
| 1. Hamid et al, (2020) | The study participants were refugees. |
| 1. Biscaglia et al,(2019) | The study participants were refugees. |
| 1. Makan et al, (2019) | The study participants were refugees. |
| 1. Salim et al, (2021) | The study participants were refugees. |
| 1. Salim et al, (2021) | The study participants were refugees. |
| 1. Høyvik et al, (2019) | The study participants were refugees. |
| 1. Riatto et al, (2018) | The study participants were refugees. |
| 1. Kazwini et al,(2021) | The study participants were refugees. |
| 1. Flynn et al, (2021) | The study participants were refugees. |
| 1. Zhang et al. (2013) | The study participants were ethnic minorities. |
| 1. Chen et al. (2021) | The study participants were ethnic minorities. |
| 1. Premaraj et al. (2020) | The study participants were ethnic minorities. |
| 1. Sgan-Cohen et al. (2014) | The study participants were ethnic minorities. |
| 1. Shi et al. (2018) | The study participants were ethnic minorities. |
| 1. Van Der Tas et al. (2016) | The study participants were ethnic minorities. |
| 1. Wu et al. (2021) | The study participants were ethnic minorities. |
| 1. Wulaerhan et al. (2014) | The study participants were ethnic minorities. |
| 1. Zhang et al. (2014) | The study participants were ethnic minorities. |
| 1. Zhang et al (2015) 2. Matsuo et al (2015) 3. Mallik et al (2012) 4. Moss et al (2023) 5. Lim E et al (2020) 6. Jardim et al (2015) 7. Owens et al (2013) 8. Drummond et al (2015) | The study participants were ethnic minorities.  The study participants were ethnic minorities.  The study participants were ethnic minorities.  The study participants were ethnic minorities.  The study participants were ethnic minorities.  The study participants were ethnic minorities.  The study participants were ethnic minorities.  The study participants were ethnic minorities. |
| 1. Stecksén-Blicks et al. (2014) | Data was collected in 2007. |
| 1. Sanders (2020) | Data was collected in 2008. |
| 1. Heinrich-Weltzien et al(2014) | Data was collected in 2005. |
| 1. Cvikl et al. (2014) | Data was collected in 2007. |
| 1. Vered Y et al. (2011) 2. Delgado-Angulo et al(2017) 3. Ghiabi et al (2013) 4. Lau et al (2012) 5. Quach et al (2015) | Data was collected in 2000.  Data was collected in 2009.  Data was collected in 2008.  Data was collected in 2003.  Data was collected in 2006. |

**Appendix 4: Comparison of caries prevalence between immigrant and control groups divided by dentition stage**


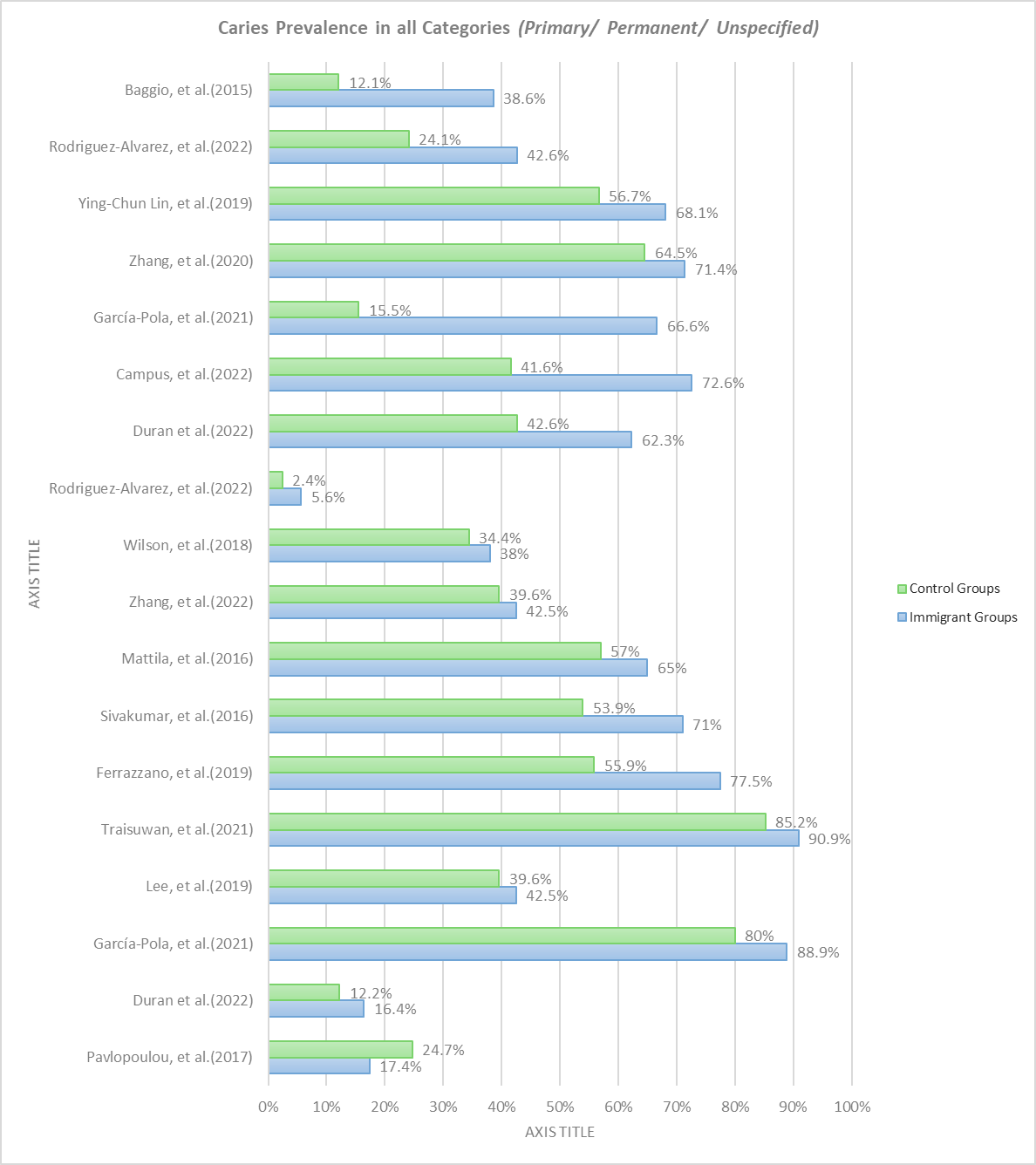


***Permanent Dentition:***

***Primary Dentition:***

***Unspecified Dentition:***

**Appendix 5: History of dental visits in immigrants.**

|  |  |  | | |  | | | | | | | | | |
| --- | --- | --- | --- | --- | --- | --- | --- | --- | --- | --- | --- | --- | --- | --- |
| ***Author*** | Christian et al,(23) | | Azrak et al,(29) | Elyasi et al,(27) | | | Meva Altas et al¸(50) | Amin et al,(26) | Dahlan et al,(28) | Traisuwan et al,(49) | | Arabi et al,(33) |  |  |
|  |  | |  | | | ***Children*** | | | |  | ***Adults*** | | |  |
| ***Percentage of participants, not visiting a dentist within last year or in their lifetime (%)*** | *88** | | *72.7** | *65.7* | | | *61.3** | *52** | *51.2* | *61.1* | | *68.9* |  |  |

*Not visiting a dentist in their lifetime.

**Appendix 6: Reference list of excluded studies**

1. Nicol P, Anthonappa R, King N, Slack‐Smith L, Cirillo G, Cherian S. Caries burden and efficacy of a referral pathway in a cohort of preschool refugee children. Aust Dent J. 2015;60(1):73-9.
2. Marwaha P, Ghanim A, Shankumar R. Risk indicators of dental caries among refugee patients attending a public dental service in Victoria. Aust Dent J. 2022;67(1):21-29.
3. Moreau A-M, Hennous F, Dabbagh B, Ferraz dos Santos B. Oral health status of refugee children in Montreal. Immigr Minor Health. 2019;21(4):693-698.
4. van Berlaer G, Carbonell FB, Manantsoa S, de Béthune X, Buyl R, Debacker M, et al. A refugee camp in the centre of Europe: clinical characteristics of asylum seekers arriving in Brussels. 2016;6(11):e013963.
5. Goetz K, Winkelmann W, Steinhäuser J. Assessment of oral health and cost of care for a group of refugees in Germany: a cross-sectional study. BMC Oral Health. 2018;18(1):69.
6. Solyman M, Schmidt-Westhausen A-M. Oral health status among newly arrived refugees in Germany: a cross-sectional study. MC Oral Health. 2018;18(1):132.
7. Takriti M, Al-Ani A, Walter M, Alkilzy M, Splieth CH. Dental status and prosthetic treatment needs among adult refugees in Germany. Quintessence Int. 2021;52(9):764-771.
8. Al-Ani A, Takriti M, Schmoeckel J, Alkilzy M, Splieth C. National oral health survey on refugees in Germany 2016/2017: caries and subsequent complications. Clin Oral Investig. 2021;25(4):2399-2405.
9. Kakalou E, Riza E, Chalikias M, Voudouri N, Vetsika A, Tsiamis C, et al. Demographic and clinical characteristics of refugees seeking primary healthcare services in Greece in the period 2015–2016: A descriptive study. Int Health. 2018;10(6):421-429.
10. Bhatt S, Gaur A. Dental caries experience and utilization of Oral Health services among Tibetan refugee-background children in Paonta sahib, Himachal Pradesh, India. J Immigr Minor Health. 2019;21(3):461-465.
11. Noaman BR, Khalid RF, Fattah LD. Maternal Dental Health Knowledge and Its Relation to the Dental Caries Experience of Their Children in Mamyzawa Camp of Refugees in Erbil, Iraq. Acta Med Acad. 2019;48(3):294-302.
12. Hamid RN, Mudher SH, Ali SM. Caries Index, Root Caries Index and Gingival Index in Immigrants at the Camp of Arbat in Sulaimaniya Governorate, Iraq. Inter Med J. 2020;27(3):357-9.
13. Biscaglia L, di Caccamo P, Terrenato I, Arrica MA, Seita A, Campus G. Oral health status and caries trend among 12-year old Palestine refugee students: results from the UNRWA’s oral health surveys 2011 and 2016. BMC Oral Health. 2019 ;19(1):157.
14. Makan R, Gara M, Awwad MA, Hassona Y. The oral health status of Syrian refugee children in Jordan: An exploratory study. Spec Care Dentist. 2019;39(3):306-309.
15. Salim NA, Alamoush RA, Al-Abdallah MM, Al-Asmar AA, Satterthwaite JD. Relationship between dental caries, oral hygiene and malocclusion among Syrian refugee children and adolescents: a cross-sectional study. BMC Oral Health. 2021;21(1):1-8.
16. Salim NA, Sawair FA, Satterthwaite JD, Al-Zubi Z. Oral Hygiene Awareness, Practices and Attitudes among Syrian Refugees in Zaatari Camp and Their Impact on Oral Health Status. Oral Health Prev Dent. 2021;19(1):689-698.
17. Høyvik AC, Lie B, Grjibovski AM, Willumsen T. Oral health challenges in refugees from the Middle East and Africa: a comparative study. J Immigr Minor Health. 2019;21(3):443-450.
18. Riatto SG, Montero J, Pérez DR, Castaño-Séiquer A, Dib A. Oral health status of Syrian children in the refugee center of Melilla, Spain. Int J Dent. 2018;2018:2637508.
19. Kazwini R, Kasem T, Alhuda NEA, Albarshah M, Subeh D, Alhaffar M. Oral health assessment in a sample of displaced people as a result of the war in Syria in Damascus city: results of non-profit initiative. BMC Oral Health. 2021;21(1):1-6.
20. Flynn PM, Petersen A, Entinger J, Shire A. The Association of Social Determinants of Health with Somali Refugee Mother–Child Caries. J Immigr Minor Health. 2021;23(3):615-23.
21. Zhang S, Liu J, Lo ECM, Chu CH. Dental caries status of dai preschool children in yunnan province, china. BMC Oral Health. 2013;13(1).
22. Chen KJ, Liu J, Xu B, Li Y, Li Y, Zhang S, et al. Oral health status of 12-year-old children in Lisu minority ethnic group in China: a cross-sectional study. BMC Oral Health. 2021;21(1):27.
23. Premaraj TS, Vella R, Chung J, Lin Q, Panier H, Underwood K, et al. Ethnic variation of oral microbiota in children. Sci Rep. 2020;10(1):14788.
24. Sgan-Cohen HD, Margvelashvili V, Bilder L, Kalandadze M, Gordon M, Margvelashvili M, et al. Dental caries among children in Georgia by age, gender, residence location and ethnic group. Community Dent Health. 2014;31(3):163-6.
25. Shi C, Faris P, McNeil DA, Patterson S, Potestio ML, Thawer S, et al. Ethnic disparities in children's oral health: findings from a population-based survey of grade 1 and 2 schoolchildren in Alberta, Canada. BMC Oral Health. 2018;18(1):1.
26. Van Der Tas JT, Kragt L, Veerkamp JJS, Jaddoe VWV, Moll HA, Ongkosuwito EM, et al. Ethnic Disparities in Dental Caries among Six-Year-Old Children in the Netherlands. Caries Research. 2016;50(5):489-97.
27. Wu SC, Ma XX, Zhang ZY, Lo ECM, Wang X, Wang B, et al. Ethnic Disparities in Dental Caries among Adolescents in China. J Dent Res. 2021;100(5):496-506.
28. Wulaerhan J, Abudureyimu A, Bao XL, Zhao J. Risk determinants associated with early childhood caries in Uygur children: a preschool-based cross-sectional study. BMC Oral Health. 2014;14:136.
29. Zhang S, Liu J, Lo ECM, Chu CH. Dental and periodontal status of 12-year-old Bulang children in China. BMC Oral Health. 2014;14(1).
30. Zhang S, Xu B, Liu J, Lo ECM, Chu CH. Dental and periodontal status of 12-yearold Dai school children in Yunnan Province, China: A cross-sectional study. BMC Oral Health. 2015;15(1).
31. Matsuo G, Rozier RG, Kranz AM. Dental caries: racial and ethnic disparities among North Carolina kindergarten students. American journal of public health. 2015 Dec;105(12):2503-9.
32. Mallik BK, Panda T, Padhy RN. Traditional herbal practices by the ethnic people of Kalahandi district of Odisha, India. Asian Pacific Journal of Tropical Biomedicine. 2012 Feb 1;2(2):S988-94.
33. Luo H, Moss ME, Wright W, Webb M, Pardi V, Lazorick S. Racial/ethnic disparities in preventive dental services use and dental caries among children. Journal of Public Health Dentistry. 2023 Mar 7.
34. Lim E, Davis J, Siriwardhana C, Aggarwal L, Hixon A, Chen JJ. Racial/ethnic differences in health-related quality of life among Hawaii adult population. Health and Quality of Life Outcomes. 2020 Dec;18:1-6.
35. Gaetti-Jardim Jr E, Pereira MF, Vieira EM, Schweitzer CM, Okamoto AC, Ávila-Campos MJ. Occurrence of periodontal pathogens in ethnic groups from a native Brazilian reservation. Archives of Oral Biology. 2015 Jun 1;60(6):959-65.
36. Fisher‐Owens SA, Isong IA, Soobader MJ, Gansky SA, Weintraub JA, Platt LJ, Newacheck PW. An examination of racial/ethnic disparities in children's oral health in the United States. Journal of Public Health Dentistry. 2013 Mar;73(2):166-74.
37. Drummond AM, Ferreira EF, Gomes VE, Marcenes W. Inequality of experience of dental caries between different ethnic groups of Brazilians aged 15 to 19 years. PloS one. 2015 Dec 22;10(12):e0145553
38. Stecksén-Blicks C, Hasslöf P, Kieri C, Widman K. Caries and background factors in Swedish 4-year-old children with special reference to immigrant status. Acta Odontologica Scandinavica. 2014 Nov 1;72(8):852-8.
39. Sanders A, Cardel M, Laniado N, Kaste L, Finlayson T, Perreira K, Sotres‐Alvarez D. Diet quality and dental caries in the Hispanic Community Health Study/Study of Latinos. Journal of public health dentistry. 2020 Jun;80(2):140-9.
40. Heinrich-Weltzien R, Walther M, Goddon I, Kühnisch J. Zahngesundheit erster Molaren bei westfälischen Migranten und deutschen Schülern. Bundesgesundheitsblatt-Gesundheitsforschung-Gesundheitsschutz. 2014;1(57):128-34.
41. Cvikl B, Haubenberger-Praml G, Drabo P, Hagmann M, Gruber R, Moritz A, Nell A. Migration background is associated with caries in Viennese school children, even if parents have received a higher education. BMC oral health. 2014 Dec;14(1):1-6.
42. Vered Y, Soskolne V, Zini A, Livny A, Sgan‐Cohen HD. Psychological distress and social support are determinants of changing oral health status among an immigrant population from Ethiopia. Community dentistry and oral epidemiology. 2011 Apr;39(2):145-53.
43. Delgado‐Angulo EK, Marcenes W, Harding S, Bernabé E. Ethnicity, migration status and dental caries experience among adults in East London. Community Dentistry and Oral Epidemiology. 2018 Aug;46(4):392-9.
44. Ghiabi E, Matthews DC, Brillant MS. The oral health status of recent immigrants and refugees in Nova Scotia, Canada. Journal of immigrant and minority health. 2014 Feb;16:95-101.
45. Lau M, Lin H, Flores G. Racial/ethnic disparities in health and health care among US adolescents. Health services research. 2012 Oct;47(5):2031-59.
46. Quach A, Laemmle-Ruff IL, Polizzi T, Paxton GA. Gaps in smiles and services: a cross-sectional study of dental caries in refugee-background children. BMC Oral Health. 2015 Dec;15:1-0.
